# Recurrence of Caries under Gold Fillings

**Published:** 1902-05-15

**Authors:** H. A. Smith

**Affiliations:** Cincinnati


### RECURRENCE OF CARIES UNDER GOLD FILLINGS.

BY H. A. SMITH, D.D.S., CINCINNATI.

Read before the Cincinnati Odontological Society.

We have heard much the past few years of the proper preparation of cavities in relation to their enamel margins. As a result of these studies in "extension for prevention" interest has been revived in the question of recurrent caries away from the margin in the bottoms of cavities that have been well filled with gold or other permanent filling material.

A notable paper bearing on the subject was read before the International Dental Congress in Paris last year by J. Choquet, professor in the Dental School of Paris. The main purpose of the paper, the author stated, was the study of the etiology of dental caries, in relation to the recurrence of decay under carefully inserted fillings in thoroughly cleansed and properly prepared cavities.

This statement brings up the question of proper cavity preparation, at least so far as the removal of softened or carious dentin is concerned. Before the establishment of the germ theory of dental caries, it was the practice with thorough operators to remove all traces of softened or discolored dentin, even at the expense of pulp exposure. Dentists of this school believed an exposed pulp carefully capped was not so liable to give after-trouble as one over which a covering of partially decalcified dentin was left. At the present time there is apparently no hesitation about leaving a layer of infected dentin over the pulp, reliance being placed upon antiseptics for complete sterilization of the infected layer. That the hasty methods now in vogue for sterilization of cavities should result in frequent failure is to be expected.

It is usual to assume that in the zone of infected dentin the bacteria are arranged in platoons in alignment, when the actual condition is that the advanced guards or pickets, so to speak, are far in advance of the main army of invasion. Sterilization would be comparatively easy if the bacteria were massed; the real difficulty is our inability to reach with our antiseptics the collection of bacteria in single tubuli beyond the so-called second layer in the carious process. Frequently in our antiseptic treatment these micro-organisms have not been disturbed in the least in their habitat; they still live, proliferate and excrete lactic acid. Quoting Prof. Choquet, who believes, as do also Galippe and Miller, that "notwithstanding all the care used in the preparation of a cavity and a subsequent filling we can never affirm there will not be a recurrence of decay." There is no other explanation he thinks of, for those cases in which fillings have been inserted for five, ten, fifteen, or even twenty years, about the walls of which no leakage has occurred, and yet under these fillings is found softened or discolored dentin which in time will cause the destruction of the pulp. It is necessary to admit in such cases that the cause is an internal one, and that it has developed as the consequence of the presence of certain micro-organisms in the tubuli of a kind of dentin which appears to the eye and touch healthy.

In order to study the morphological and functional changes that occur in bacteria of caries found under fillings that have been placed for several years, Choquet instituted a series of experiments of inoculation.

From three perfectly filled teeth he succeeded in isolating five species of micro-organisms. A pure culture was obtained from one of these teeth—a lower molar which had been filled for seven years on the buccal surface, and which was extracted to prevent trouble accompanying the difficult eruption of a third molar. Choquet describes this species as a bacillus of slow development with a tendency to grow best without the presence of oxygen. With a pure culture of this bacteria he proceeded to produce dental caries in living animals. Heretofore all attempts to produce artificial caries have been upon teeth out of the mouth, and notwithstanding that specimens of artificial caries have been frequently shown by Miller and others that could not be distinguished from natural caries, there have all along been those who rejected the chemico-microbic theory of dental caries, because the specimens experimented with lacked the life element. The late Dr. Atkinson said "There is no one of those cases that can not be discerned in an instant as to which is natural and which is artificial."

In these experiments of inoculation the sheep was selected because of the docility of the animal, and also the resemblance of the animal's anterior teeth to the human incisors. One of these experiments Choquet describes—a cavity was carefully made in the labial surface of a central incisor 3MM in depth. A small drop of bouillon which had been inoculated twenty-four hours with the pure culture was deposited in the bottom of the cavity and covered with a thin platinum cup. The cavity was then filled with cement. All precautions were observed throughout the operation to maintain an asceptic condition. Nine months afterwards the animal was slaughtered and the following phenomena were observed: "The dentin, instead of being white as it normally is, was of a yellowish hue and was also softened. The softening, reaching a slight depth, was very plain and more noticeable at the portion of the cavity where the diameter had increased." A small portion of this softened dentin was removed, and on inoculating the same kind of media as originally used the same species used for the original inoculation was recognized. "Thus was demonstrated the possibility of producing artificial caries in a living animal."

It will be seen that these investigations of Choquet are unique and a study of them in detail as given in the original paper can not fail to be useful to the dentist in his every-day practice. I will briefly relate a case which occurred recently in my own practice in which Choquet's investigations and conclusions were especialy helpful in diagnosis. The case was one of obscure facial neuralgia. The patient, twenty-five years of age, seemingly in the best of health, applied to be relieved of pain of an intermittent character which she thought might be associated with some lesion of the teeth. A careful examination of the teeth on the side affected showed three superficial cavities, and these I at once filled permanently. An approximal, somewhat deep-seated cavity for prudential reasons I filled with gutta-percha. Believing the pain was not due to pulp irritation by reason of these cavities, I prescribed quinin in tonic doses, two grains three times a day for six days. The paroxysms were not so frequent and the pain was less severe during the quinin treatment. In a few days, however, severe intermittent pains returned. At this stage of the case my attention was centered upon a lower second molar which had been filled on the occlusal surface with gold five or six years before. The character of the filling was excellent, and notwithstanding that there was not the slightest evidence of recurrent caries about the margins of the cavity, I decided to remove the filling to see if there was not recurrent caries under it. I found the cavity of medium depth and the dentin in the bottom for two-thirds its extent was hard and natural in color; the remaining one-third in a labial direction was softened and slightly discolored, thus clearly showing the presence of dental caries. I found the disease had advanced lingually under the hard area I have mentioned. The pulp was not exposed. Carefully removing the softened dentin down to a layer sufficient for pulp protection, I applied antiseptic treatment. Oil of cloves and carbolic acid in the proportion of three to one, were used. Two dressings were made in seven or eight days, care being taken to seal in each. The cavity within the cavity was then filled with the softer variety of gutta-percha, care being used to avoid pressure on the pulp. After using a cavity varnish the main cavity was filled with oxyphosphate. Now after a lapse of several weeks all neuralgic pains have disappeared. The tooth when last seen responded to the tests for a living pulp.

It being granted in this case that the original filling hermetically sealed the cavity, that there was no negligence in its preparation, and that the usual custom of wiping the cavity with an antiseptic was observed, we naturally conclude that recurrent caries was due to intertubular infection such as I have described. Upon this hypothesis it took five years after the filling was inserted for caries to advance sufficiently near the pulp to cause disturbance. The phenomena observed in the case I have related tends to confirm Choquet's conclusion that "the destructive process of caries may take place under good fillings of whatever material after the lapse of a long time.''

The paper which suggested the title of my own, and from which I have quoted, was one of the two or three which were read before the Paris Congress that attracted special attention. I would earnestly recommend it for careful study. I am aware the thoughtless dentist will say it is too scientific and ask of what practical benefit is all this? In answer I beg to remind him—The successful *practice* of dentistry is always based on the *science* of dentistry.

DISCUSSION.

Dr. J. R. Callahan: It is almost impossible to sterilize the substance within the pulp-chamber, and in those cavities where we leave decay I am sure the infection reaches clear to the pulp. The preparation of the cavity is of really more importance than the filling.

Dr. J. S. Cassidy: I should name this paper ''The Failure of Good Fillings.'' Otherwise we might suppose the failure was due to some defect in the preparation of the cavity. As it is, we must suppose the cavity properly prepared, all infected matter removed, and the cavity properly filled. Further, we must restrict ourselves to destructive influences that would bring about decay. These are in the nature of possibilities of attack proceeding from outside the filling itself. We have suggested that recurring caries may come on the surface of fillings as well as under them. In caries proceeding from the surface near the filling we have conditions which must be considered similar to those existing before the teeth decayed at all, but we have in connection with that condition the presence of the foreign body--the filling. If these fillings be foreign in their physical properties—amalgam and gold—we must note that the difference between the two substances and the potentiality in specific heat and other innate properties figure in recurring caries. We do not see decay recurring as a rule on the surfaces of non-metallic fillings. The combining of amalgam and gold in a filling results in the immediate polarizing of the two metals, which is unfavorable to the recurrence of caries. It occurs to me that neurasthenia may influence decay of the teeth. If through nervous perversion such phenomena as absorption of tissues may result, with an accompaniment of excretion of lactic acid, why may not the same thing occur in the pulp of the teeth, the accompanying generation of lactic acid accounting for the recurrence internally of caries under even a perfect filling.

Dr. O. N. Heise: This last suggestion is certainly novel, and perhaps Dr. Cassidy has made a new discovery. We are taught that the pulp is capable of throwing out secondary dentin, so why may it not secrete lactic acid? I fear, however, that if this theory should be substantiated it would discourage care in filling teeth. Dr. Choquet investigating along lines similar to those suggested by Dr. Smith's paper, made experiments to show decay spontaneously re-asserting itself under circumstances much like those given in the paper. From the details which he gives, however, I am inclined to think that he did not conform closely enough to the conditions laid down to entitle his conclusions to serious consideration. With a cavity carefully excavated and filled I do not think internal decay can occur.

Dr. H. A. Smith: If I intimated that there is no therapeutic value in the filling material I went too far. Dr. Miller's experiments show that copper amalgam prevents to a great extent the growth of micro-organisms, but the same is not true of cohesive gold. The theory I have advanced to account for recurrence of decay under perfect fillings finds its analogue in the cases of plants transferred from a tropical to a temperate zone. They may continue to grow, but not luxuriantly, and the micro-organisms in the dentin of the teeth develop very slowly indeed, but still they develop. We arrest decay but we do not prevent it.—*Digest.*

